# Supplementary figures and images for: Do Brain Networks Evolve by Maximizing Their Information Flow Capacity?
Source: PLoS Comput Biol. 2015 Aug 28;11(8):e1004372. doi: 10.1371/journal.pcbi.1004372 (PMC4552863; doi:10.1371/journal.pcbi.1004372)

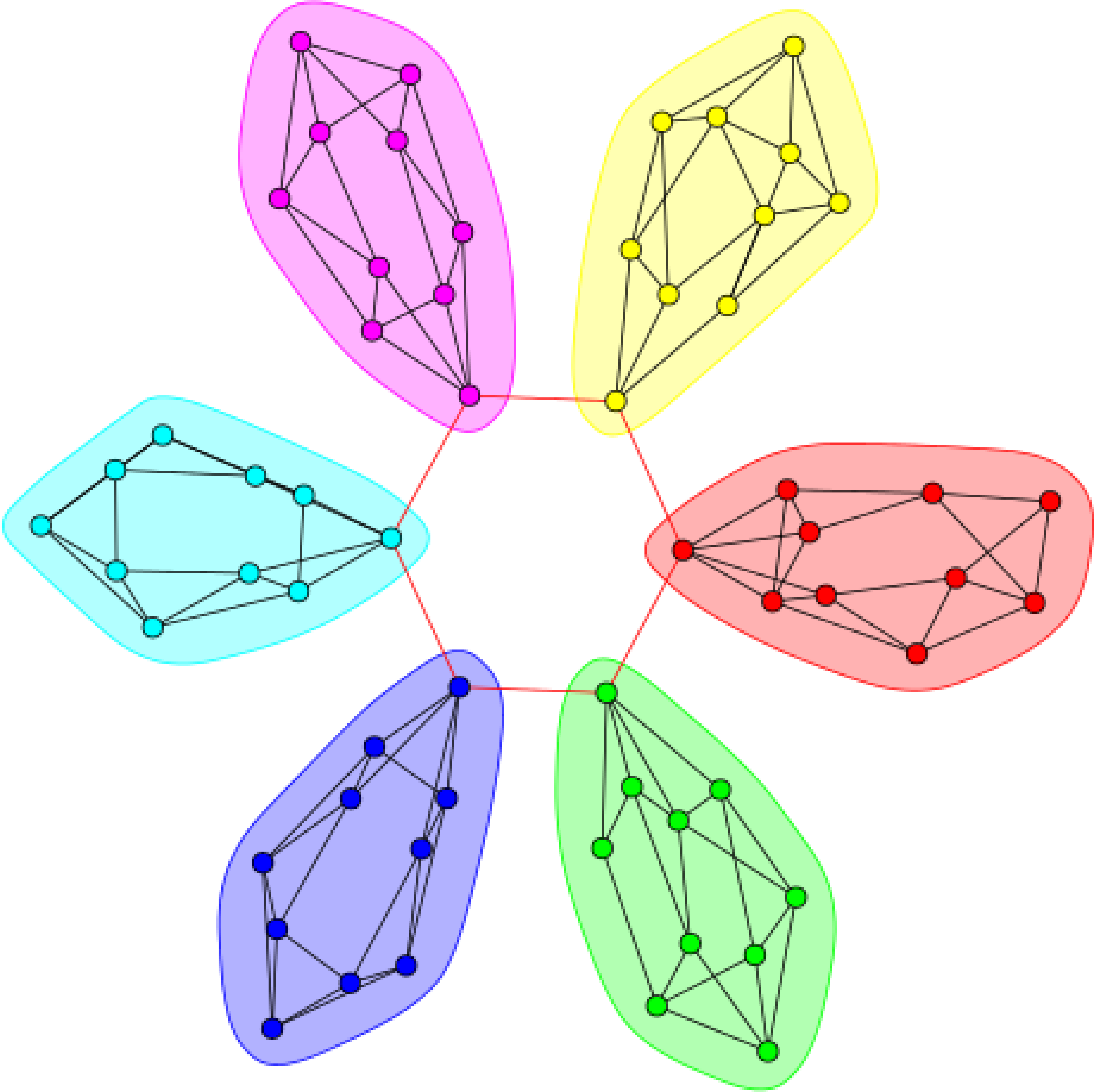

Supplement: S1 Fig — It comprises N n = 60 neurons arranged in N c = 6 small-world clusters. The red ring consists of chemical excitatory connections that link all hubs of the network. Within each cluster, depicted by a differently color-shaded area of intra-connected neurons, we consider solely electrical connections denoted by black edges. (TIF) [file pcbi.1004372.s001.tif]

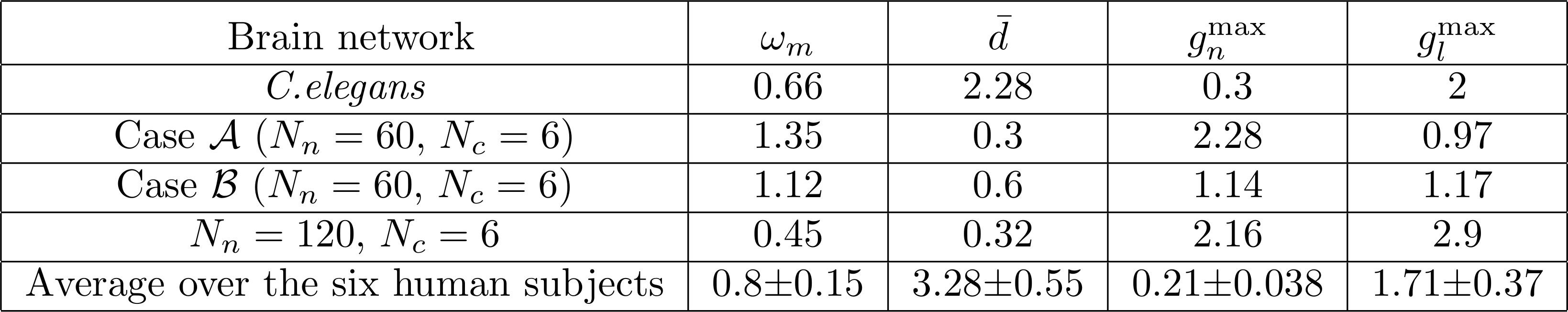

Supplement: S1 Table — These values were used to provide rough estimates for the extend of the couplings of the parameter spaces, gnmax and glmax, based on those of the zoomed-in parameter space of the C.elegans of Fig 2 of the main manuscript (first row of the Table). (TIF) [file pcbi.1004372.s003.tif]

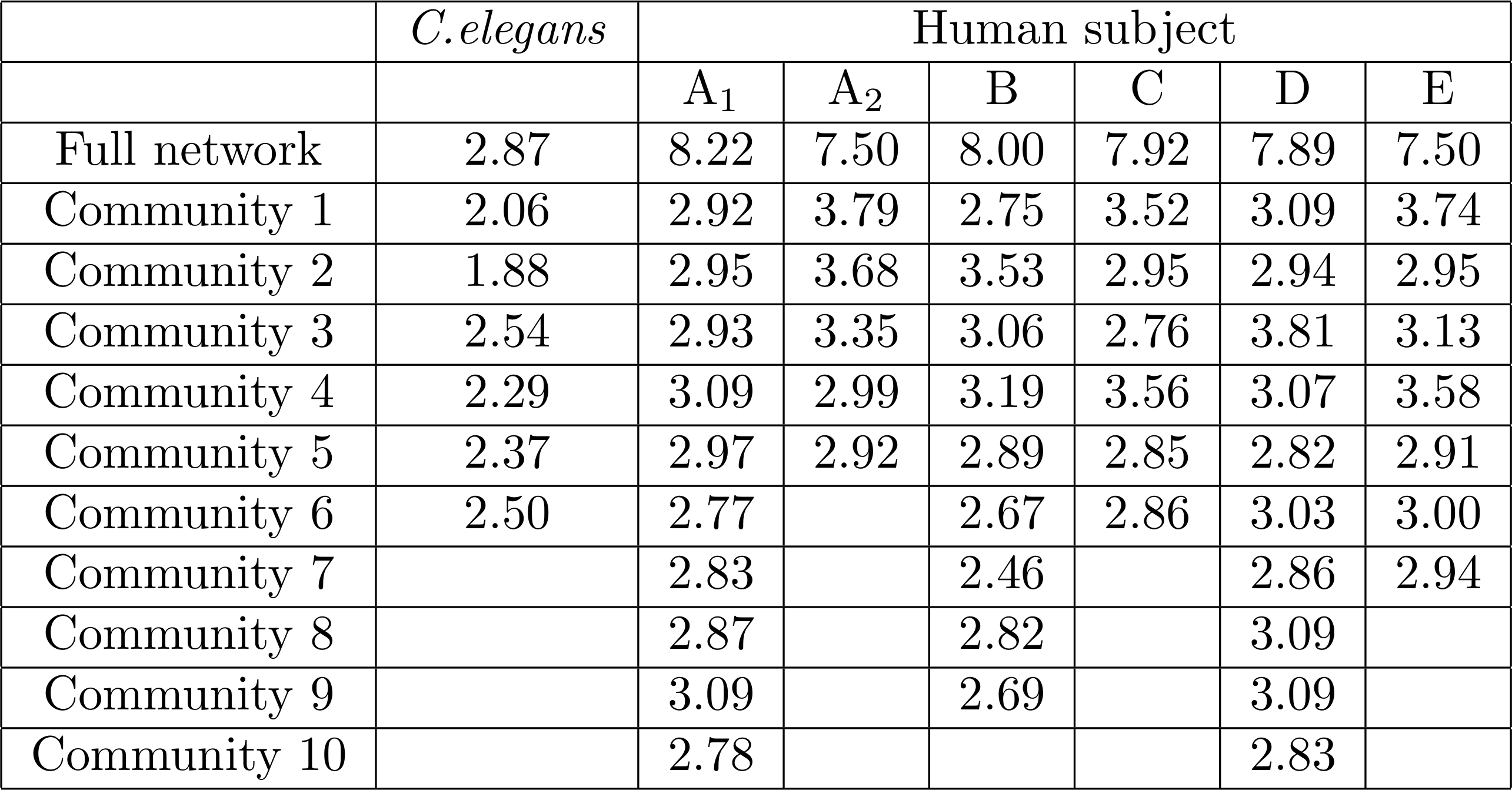

Supplement: S2 Table — (TIF) [file pcbi.1004372.s004.tif]
